# Reduced unilateral sweating caused by varicella zoster virus infection: a case report

**DOI:** 10.1186/s12883-024-03770-5

**Published:** 2024-07-24

**Authors:** Wenju Li, Bingquan Leng, Jing Zhao, Yu Zhang, Lili Yu, Chang Liu, Kun Hong

**Affiliations:** 1 Department of Neurology, Central Hospital of Rizhao, 66 Wanghai Road, Rizhao Shandong, 276800 China; 2 Department of Neurology, Second Hospital of Hebei Medical University, Shijiazhuang City, Hebei Province China; 3 Neurological Laboratory of Hebei Province, Shijiazhuang, China

**Keywords:** Varicella-Zoster virus (VZV), Herpes zoster (HZ), Autonomic dysfunction, Viral meningitis, Sympathetic skin response (SSR), Next-generation sequencing (NGS)

## Abstract

**Background:**

Herpes zoster is an infectious skin disease caused by the reactivation of the varicella zoster virus (VZV), which has been latent in the posterior root ganglia of the spinal cord or cranial ganglia for an extended period. Neurological complications caused by herpes zoster include aseptic meningitis, white matter disease, peripheral motor neuropathy, and Guillain-Barré syndrome. However, reduced unilateral sweating caused by the VZV is very rare.

**Case Presentation:**

This article reports the case of a 34-year-old woman who was admitted to our hospital with sore throat, dizziness, and reduced sweating on the left side of her body. Physical examination found herpes lesions on the left upper lip and left external ear canal (scabbed) and reduced sweating on the left side of the body. Head magnetic resonance imaging (MRI) with contrast showed no abnormalities. After a lumbar puncture, the patient was diagnosed with viral meningitis by VZV infection. The electromyographic skin sympathetic reflex indicated damage to the left sympathetic nerve.

**Conclusions:**

Secondary unilateral sweating reduction is a rare neurological complication of herpes zoster, caused by damage to the autonomic nervous system. Literature review and comprehensive examination indicated that the reduced unilateral sweating was due to the activation of latent herpes zoster virus in the autonomic ganglia which has damaged the autonomic nervous system. For patients who exhibit acute hemibody sweat reduction, doctors should consider the possibility of secondary autonomic nervous system damage caused by herpes zoster.

**Supplementary Information:**

The online version contains supplementary material available at 10.1186/s12883-024-03770-5.

## Background

Varicella-zoster virus (VZV) is a human-specific neurotropic herpes simplex virus. The primary infection causes chickenpox, and the virus lurks along the entire nerve axis in the posterior root ganglia of the spinal cord or cranial ganglia [1]. When reactivated, it usually manifests as herpes zoster (HZ) [2]. A common symptom of HZ is painful skin rashes often occurring in the intercostal, cervical, trigeminal, and lumbosacral dermatomes [3].VZV reactivation can also present as central nervous system infections [4], such as transverse myelitis and encephalitis [5]. Herpes zoster (HZ) rarely causes a decrease in unilateral sweating, and there is currently a limited understanding of this condition. Herein, we report a case of HZ infection that led to reduced unilateral sweating. We provide a pertinent review of the literature. However, further research is needed regarding the location of the infection and the potential mechanisms of its damage. Therefore, this article presents a case report and reviews the relevant literature.

### Case presentation

**Fig. 1:**
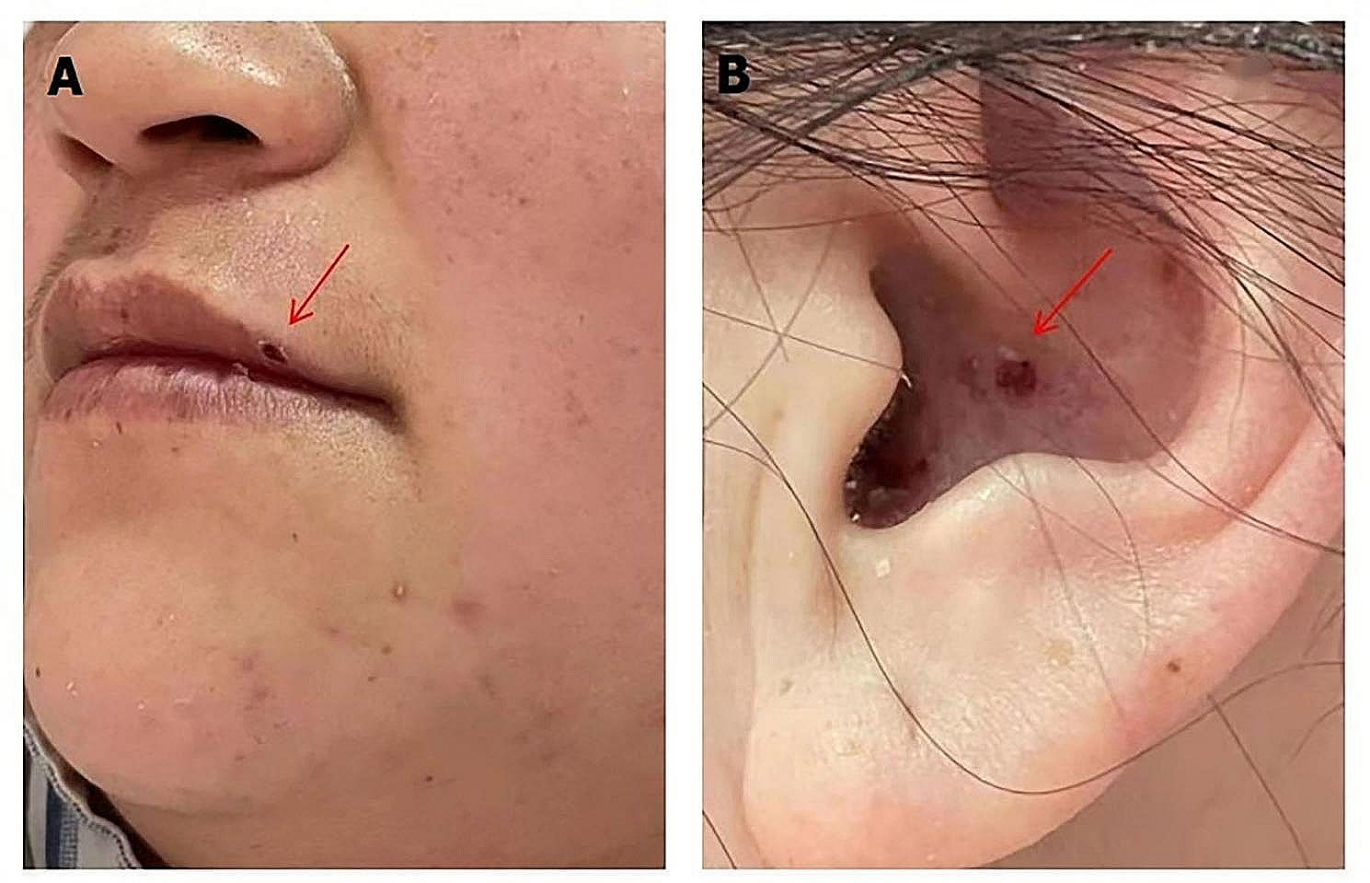
Multiple blisters (scabbed) are visible on the skin of the patient’s left upper lip (**A**) and left external ear canal (**B**)

**Fig. 2:**
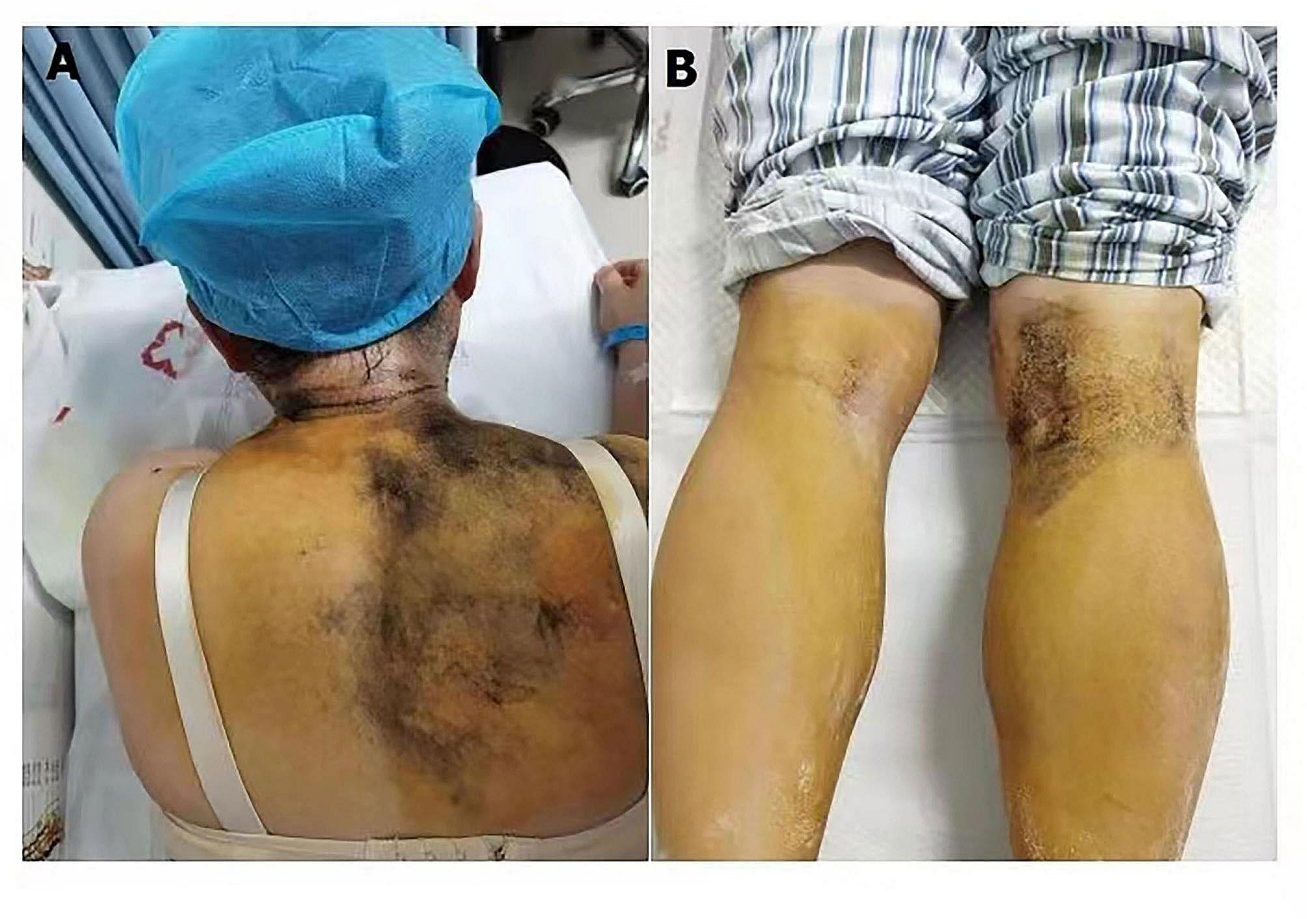
We conducted an improved Minor’s iodine-starch test, which showed sweating in the right back and reduced sweating in the left back (**A**), and sweating in the right popliteal fossa and reduced sweating in the left popliteal fossa (**B**)

A 34-year-old woman was admitted to our hospital because of sore throat, radiating pain in the left cheek and back of the ear for 10 days, fever (a maximum body temperature of 38 °C), dizziness, and reduced sweating on the left side of the body for 9 days. She reported unstable walking, nausea, and poor appetite and had developed a herpes infection on the left upper lip and left external ear canal for 9 days. However, the patient did not experience symptoms such as vomiting, headache, joint pain, hoarseness, or difficulty in swallowing. The patient received antiviral treatment with acyclovir administered intravenously (10 mg/kg, q8h) for more than 7 days; however, there was no improvement in the symptoms. Physical examination showed a vesicular rash over the left lip and left external auditory canal (scabbed), as shown in Fig. 1A and B, decreased sweating on the left side of the body, except the face, as shown in Fig. 2A and B, soft neck, Kernig’s sign (-) and Brudzinski’s sign (-). Mental status examination, cranial nerves, motor exam, reflexes, sensory testing, coordination, and gait were normal.

**Fig. 3:**
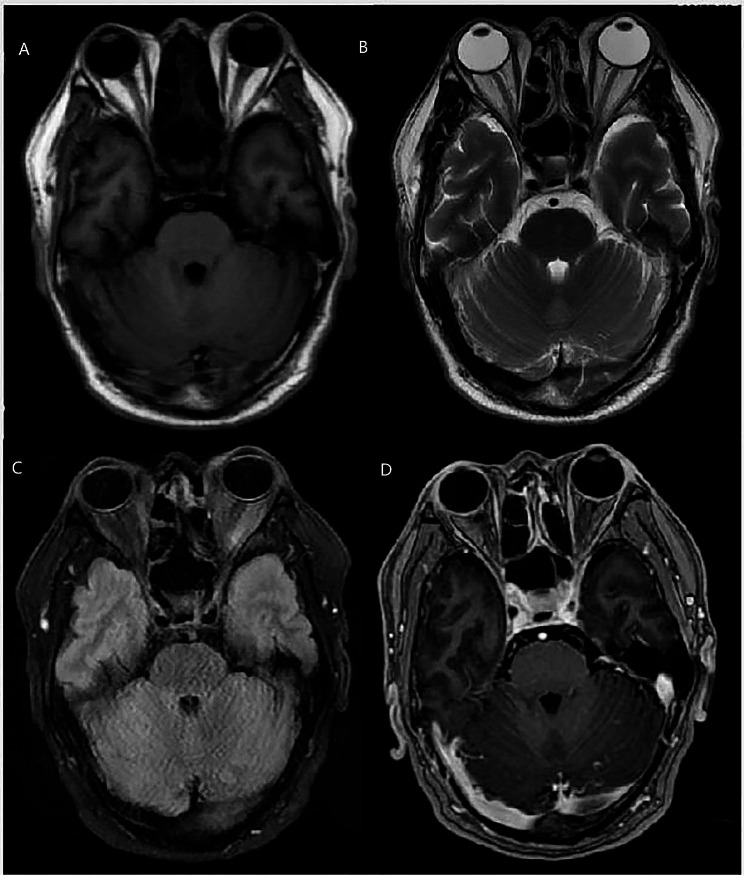
Head MRI with contrast showed no abnormalities

We conducted a Minor’s iodine-starch test, which showed reduced sweating in the left posterior back and left popliteal fossa (Fig. 2). The Minor’s iodine-starch test was performed by staining the patient with an iodine solution (1.5 g iodine, 10 g castor oil, and 125 ml 95% ethanol) [6]. Head magnetic resonance imaging (MRI) with contrast showed no abnormalities (Fig. 3). Based on the sore throat, dizziness, fever, reduced sweating on the left side of the body, and herpes infection on the left upper lip and left external ear canal, we considered the possibility of VZV meningitis; therefore, a lumbar puncture was performed.

**Fig. 4:**
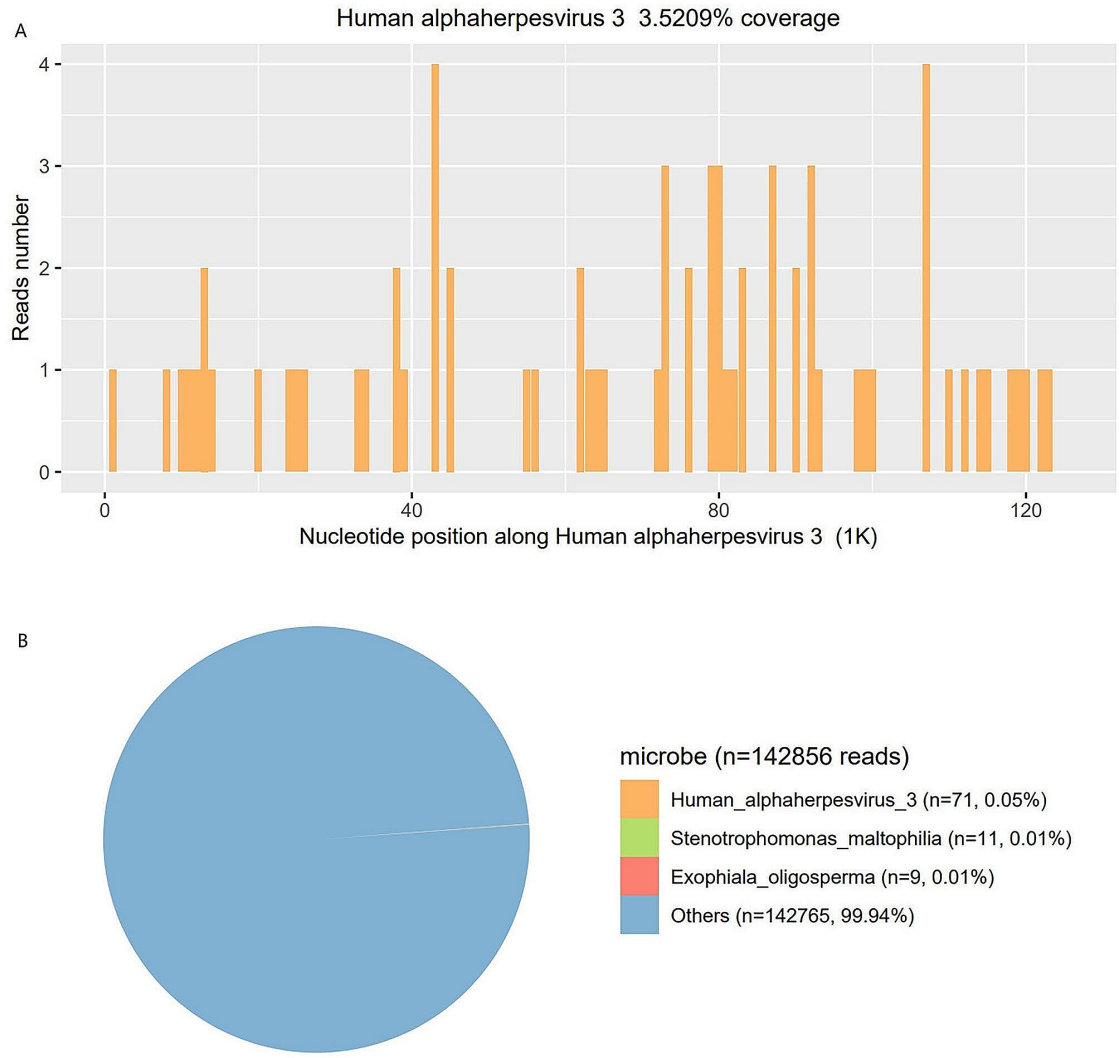
mNGS of Human alphaherpesvirus-3 in the patient’s cerebrospinal fluid (**A**), and (**B**) the result of mNGS showed 71 reads corresponding to the human alphaherpesvirus-3, with a coverage of 3.5209%. In the microbiota composition, 0.05% of the reads corresponded to human alphaherpesvirus-3, and the remainder are commonly regarded as contaminating bacterial DNA from the environment

The cerebral spinal fluid (CSF) opening pressure was 105 mmH_2_O. Routine and biochemical testing of CSF identified the following: protein 0.17 g/L (0.20–0.40); leukocytes 26 × 10^6^ /L (0.0–15.0); glucose 58.5 mg/dl (45–81). CSF cytology was mainly characterized by lymphocyte reactions; we did not find atypical cells, ruling out lymphoma. CSF pathogenic examination showed a negative CSF culture. Next-generation sequencing (NGS) of CSF was used for the detection of pathogens. After 48 h, the results revealed 71 sequence reads uniquely corresponding to the VZV genome with 3.5209% coverage (Fig. 4).

**Fig. 5:**
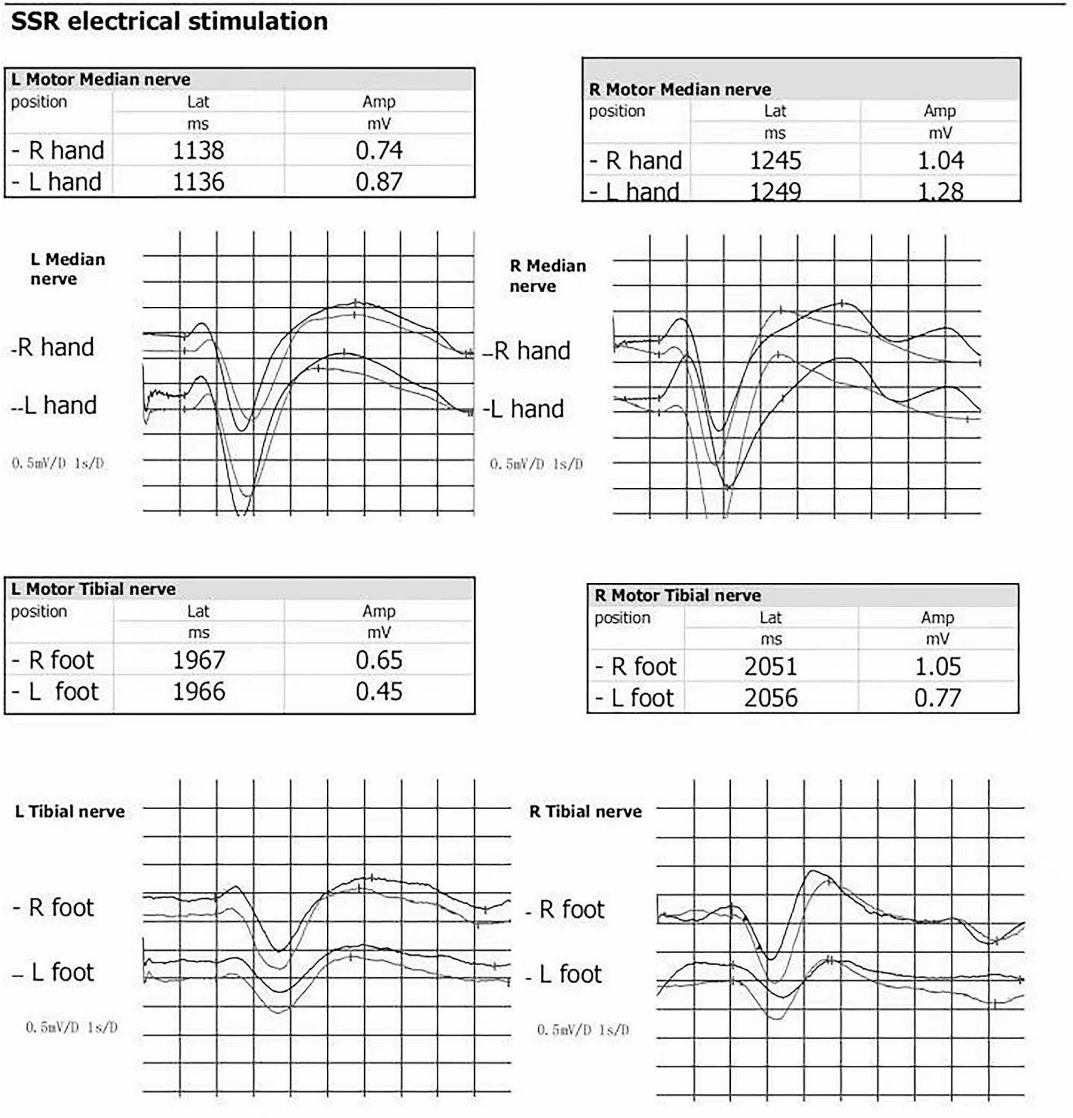
SSR for the hands and feet. The latency and amplitude of both hands and feet are within the normal range. The amplitude obtained by stimulating the left hand is slightly lower than that obtained by stimulating the right hand. The amplitude obtained by stimulating the left foot is slightly lower than that obtained by stimulating the right foot (Normal values: arm latency ≤ 1512 ms, arm amplitude ≥ 0.484 mV; leg latency ≤ 2230 ms, leg amplitude ≥ 0.364 mV)

Sympathetic skin response (SSR): The amplitude obtained by stimulating the left hand was slightly lower than that obtained by stimulating the right hand. The amplitude obtained by stimulating the left foot was slightly lower than that obtained by stimulating the right foot. SSR shows left-sided autonomic nerve damage, as shown in Fig. 5.

The patient was diagnosed with viral meningitis by VZV infection and multiple cranial nerve damage (trigeminal and vestibular nerves). We administered 3 g of sodium phosphonate as an antiviral along with B-vitamin nutritional therapy. The role of sodium phosphonate as an antiviral involves blocking the phosphate binding site of viral DNA polymerase in a non-competitive manner to prevent the viral DNA strands extension [7]. After one week, a repeat lumbar puncture was performed, with the CSF test showing a CSF pressure of 120 mmH_2_O, cerebrospinal fluid white blood cell count of 1 cell/mm^3^, and a CSF protein level of 8 mg/dl. The patient recovered well from the sore throat, with dizziness disappearing and resumption to a normal body temperature. Two months after discharge, the reduced sweating on the left side of the body returned to normal. The one-year follow-up after discharge showed no significant sequelae.

## Discussion and conclusions

HZ, also known as zoster, is an infectious skin disease caused by the reactivation of the VZV, which has been dormant in the posterior root ganglia or cranial ganglia of the spinal cord [8]. HZ is a common viral skin infection [9].

In addition to skin damage, HZ is often accompanied by neuropathic pain and is more common in older, immunosuppressed, and immunodeficient populations. It can seriously impact patient quality of life [6], and many patients experience severe physical, occupational, social, and psychosocial disabilities due to persistent pain. Reports in the literature suggest that VZV can also cause HZ in the eyes, which can be complicated by corneal perforation, acute iridocyclitis, vitreous inflammation, necrotizing retinitis, and obstructive retinal vasculitis, potentially leading to retinal detachment, decreased vision, and blindness [10]. HZ is often accompanied by dizziness and changes in taste and hearing. Neurological complications include aseptic meningitis, white matter disease, peripheral motor neuropathy, and Guillain-Barré syndrome [11]. Patients with severe immune deficiency are prone to disseminated HZ and visceral damage, which can manifest as pneumonia, hepatitis, or encephalitis [12].

In this case, we found a VZV infection that caused a decrease in unilateral sweating. This sequela is very rare, and further research is needed on the location and potential mechanisms of its damage. The SSR examination showed a decrease in the left side of wave amplitude, as shown in Fig. 5. This indicated that the patient had autonomic nerve damage on the left side. To further clarify the potential mechanisms of this damage, we examined the literature.

It is known that the VZV lurks in the brain ganglia and spinal dorsal root ganglia, but it can also remain dormant in autonomic ganglia [13], a finding which is rarely documented. In 2001, researchers used polymerase chain reaction (PCR) to detect the DNA of the VZV in human lymph nodes and abdominal ganglia, the first detection of VZV DNA in human autonomic nervous system ganglia [14]. The pathological and physiological mechanisms of autonomic nerve injury remain unclear. Some studies report that reactivation of the virus in the posterior root ganglia and its transmission through affected nerves leads to severe ganglial inflammation and neuritis. This process results in strong sympathetic nerve stimulation and vasoconstriction of small arteries within the nerve, thereby reducing blood flow in the capillary bed within the nerve and causing nerve ischemia [15]. It can therefore be inferred that the virus can reactivate in the autonomic ganglia, spread through the affected nerves, and damage the autonomic nervous system. The patient may have a weakened immune system after catching a cold, and the VZV in the brain ganglia and autonomic ganglia may be activated, damaging the glossopharyngeal, vestibular, trigeminal, autonomic, and retrograde nerve roots entering the cerebrospinal fluid, resulting in the various discomfort symptoms mentioned earlier.

There are few reports of VZV infection complicated by damage to the autonomic nervous system. In 2022, researchers systematically reviewed 45 articles on autonomic dysfunction caused by VZV infection since 1956, including four cases of pupillary dysfunction, two of uterine dysfunction, two of cardiovascular dysfunction, 14 of gastrointestinal dysfunction, and 23 of urinary and reproductive dysfunction [5]. There is only one case reported of reduced sweating caused by VZV infection [16]. However, that study did not confirm autonomic nervous system damage through examination, nor did it mention the potential mechanisms of damage to the autonomic nervous system. With this case report, we demonstrate that VZV infection can lead to unilateral autonomic nervous system damage, and we have attempted to clarify the potential mechanisms of its damage.

Because the symptoms of limb pain may mask the symptoms of reduced lateral sweating, the incidence of secondary reduced lateral sweating in HZ may be underestimated. We aimed to raise general awareness of this symptom. However, VZV infection causing unilateral autonomic nerve damage has rarely been described as a neurological complication of HZ. The significance of this case lies in demonstrating the need for dermatologists to understand and identify the syndrome so that antiviral treatment can be initiated early to avoid other possible serious complications. It should also be of value in multidisciplinary patient management with neurologists and/or otolaryngologists.

In summary, the secondary reduction of unilateral sweating is a rare neurological complication of HZ, and the mechanism of its occurrence has not yet been elucidated. At present, there is a lack of evidence-based medicine for treatment; however, in this report, the patient’s antiviral treatment was effective, and the prognosis was relatively good. For patients with acutely reduced sweating, physicians should consider the possibility of secondary autonomic nervous system damage caused by HZ. Future research should investigate the underlying mechanism of the symptoms noted here.

### Electronic supplementary material

Below is the link to the electronic supplementary material.

Supplementary Material 1

Supplementary Material 2

## Data Availability

No datasets were generated or analysed during the current study.

## Abbreviations

CSF: Cerebral spinal fluid
HZ: Herpes zoster
MRI: Magnetic resonance imaging
NGS: Next-generation sequencing
PCR: Polymerase chain reaction
RHS: Ramsay Hunt syndrome
SSR: Sympathetic skin response
VZV: Varicella zoster virus
