# Supplementary material for: Reduced unilateral sweating caused by varicella zoster virus infection: a case report
Source: BMC Neurol. 2024 Jul 24;24:257. doi: 10.1186/s12883-024-03770-5 (PMC11267665; doi:10.1186/s12883-024-03770-5)
Supplement: Supplementary file 1 — Supplementary Material 1 [file 12883_2024_3770_MOESM1_ESM.pdf]

CARE case report guidelines

CARE Checklist of information to include when writing a case report

(cc) BY-NC-ND

Topic	Item	Checklist item description	Reported on Line
Title	1	The diagnosis or intervention of primary focus followed by the words "case report"	Page 1, line 1
Key Words	2	2 to 5 key words that identify diagnoses or interventions in this case report, including "case report"	Page 3, line 41-42
Abstract (no references)	3a	Introduction: What is unique about this case and what does it add to the scientific literature?	Page 2, line 23-24
	3b	Main symptoms and/or important clinical findings	Page 2, line 25-29
	3c	The main diagnoses, therapeutic interventions, and outcomes	Page 2, line 29-32
	3d	Conclusion—What is the main "take-away" lesson(s) from this case?	Page 2, line 37-39
Introduction	4	One or two paragraphs summarizing why this case is unique (**may include references**)	Page 3, line 51-56
Patient Information	5a	De-identified patient specific information.	Page 3, line 59
	5b	Primary concerns and symptoms of the patient.	Page 3, line 60-63
	5c	Medical, family, and psycho-social history including relevant genetic information	Page 4, line 63-65
	5d	Relevant past interventions with outcomes	Page 4, line 65-66
Clinical Findings	6	Describe significant physical examination (PE) and important clinical findings.	Page 3-4, line 59-71
Timeline	7	Historical and current information from this episode of care organized as a timeline	Page 3-4, line 59-71
Diagnostic Assessment	8a	Diagnostic testing (such as PE, laboratory testing, imaging, surveys).	Page 4-5, line 72-92
	8b	Diagnostic challenges (such as access to testing, financial, or cultural)	Page 5, line 80-92
	8c	Diagnosis (including other diagnoses considered)	Page 5, line 93-94
	8d	Prognosis (such as staging in oncology) where applicable	Page 6, line 101-103
Therapeutic Intervention	9a	Types of therapeutic intervention (such as pharmacologic, surgical, preventive, self-care)	Page 5, line 94-95
	9b	Administration of therapeutic intervention (such as dosage, strength, duration)	Page 5-6, line 94-100
	9c	Changes in therapeutic intervention (with rationale)	Page 5, line 95-97
Follow-up and Outcomes	10a	Clinician and patient-assessed outcomes (if available)	Page 5-6, line 98-103
	10b	Important follow-up diagnostic and other test results	Page 5-6, line 98-103
	10c	Intervention adherence and tolerability (How was this assessed?)	Page 5-6, line 98-103
	10d	Adverse and unanticipated events	Page 6, line 102-103
Discussion	11a	A scientific discussion of the strengths AND limitations associated with this case report	Page 6-9, line 120-160
	11b	Discussion of the relevant medical literature **with references**.	Page 11-13, line 211-257
	11c	The scientific rationale for any conclusions (including assessment of possible causes)	Page 9, line 162-168
	11d	The primary "take-away" lessons of this case report (without references) in a one paragraph conclusion	Page 9, line 162-168
Patient Perspective	12	The patient should share their perspective in one to two paragraphs on the treatment(s) they received	Page 6, line 102-103
Informed Consent	13	Did the patient give informed consent? Please provide if requested	Yes ☑ No ☐
