# Supplementary material for: Reduced unilateral sweating caused by varicella zoster virus infection: a case report
Source: BMC Neurol. 2024 Jul 24;24:257. doi: 10.1186/s12883-024-03770-5 (PMC11267665; doi:10.1186/s12883-024-03770-5)
Supplement: Supplementary file 2 — Supplementary Material 2 [file 12883_2024_3770_MOESM2_ESM.docx]

Reduced unilateral sweating caused by varicella zoster virus infection: A case report

Wenju Li^a†^, Bingquan Leng^a†^, Jing Zhao^b,c^, Yu Zhang^b,c^，Lili Yu^b,c^, Chang Liu^b,c,#^, Kun Hong^b,c,#^

^a^Department of Neurology, Central Hospital of Rizhao, 66 Wanghai Road, Rizhao Shandong. 276800，China

^b^Department of Neurology, Second Hospital of Hebei Medical University, Shijiazhuang City, Hebei Province, China

^c^Neurological Laboratory of Hebei Province, Shijiazhuang, China.

^†^ Wenju Li and Bingquan Leng have contributed equally to this work.

^#^Address correspondence to:

Kun Hong. Department of Neurology, Second Hospital of Hebei Medical University, China. E-mail：[28403129@hebmu.edu.cn](mailto:28403129@hebmu.edu.cn). Tel.: +86-311-66007336

Chang Liu. Department of Neurology, Second Hospital of Hebei Medical University, China. E-mail：28302489@hebmu.edu.cn. Tel.: +86-311-66003711

ORCID：hongkun999@163.com

**Abstract**

**Background:** Herpes zoster is an infectious skin disease caused by the reactivation of the varicella zoster virus (VZV), which has been latent in the posterior root ganglia of the spinal cord or cranial ganglia for an extended period. Neurological complications caused by herpes zoster include aseptic meningitis, white matter disease, peripheral motor neuropathy, and Guillain-Barré syndrome. However, reduced unilateral sweating caused by the VZV is very rare.

**Case Presentation:** This article reports the case of a 34-year-old woman who was admitted to our hospital for nine days with sore throat, dizziness, and reduced sweating on the left side of her body. Physical examination found herpes lesions on the left upper lip and left external ear canal (scabbed) and reduced sweating on the left side of the body. Head magnetic resonance imaging (MRI) with contrast showed no abnormalities. After a lumbar puncture, the patient was diagnosed with viral meningitis by VZV infection. The electromyographic skin sympathetic reflex indicated damage to the left sympathetic nerve.

**Conclusions:** Secondary unilateral sweating reduction is a rare neurological complication of herpes zoster, caused by damage to the autonomic nervous system. Literature review and comprehensive examination indicated that the reduced unilateral sweating was due to the activation of latent herpes zoster virus in the autonomic ganglia which has damaged the autonomic nervous system. For patients who exhibit acute hemibody sweat reduction, doctors should consider the possibility of secondary autonomic nervous system damage caused by herpes zoster.

**Keywords:** Varicella-zoster virus (VZV), herpes zoster (HZ), autonomic dysfunction, viral meningitis, sympathetic skin response (SSR), next-generation sequencing (NGS)

**Background**:

Varicella-zoster virus (VZV) is a human-specific neurotropic herpes simplex virus. The primary infection causes chickenpox, and the virus lurks along the entire nerve axis in the posterior root ganglia of the spinal cord or cranial ganglia [1]. When reactivated, it usually manifests as herpes zoster (HZ) [2]. A common symptom of HZ is painful skin rashes often occurring in the intercostal, cervical, trigeminal, and lumbosacral dermatomes [3].VZV reactivation can also present as a central nervous system infection [4], e.g. Ramsay Hunt syndrome (RHS) [5]. Herpes zoster (HZ) rarely causes a decrease in unilateral sweating, and there is currently a limited understanding of this condition. Herein, we report a case of HZ infection that led to reduced unilateral sweating. We provide a pertinent review of the literature. However, further research is needed regarding the location of the infection and the potential mechanisms of its damage. Therefore, this article presents a case report and reviews the relevant literature.

**Case presentation**

A 34-year-old woman was admitted to our hospital because of sore throat，radiating pain in the left cheek and back of the ear x 10 d，fever (a maximum body temperature of 38 °C), dizziness, and reduced sweating on the left side of the body x 9 d. She reported unstable walking, nausea, and poor appetite, and had developed a herpes infection on the left upper lip and left external ear canal x 9 d. However, the patient did not experience symptoms such as vomiting, headache, joint pain, hoarseness, or difficulty in swallowing. The patient received antiviral treatment with acyclovir (10 mg/kg, q8h, ivgtt) over one wk**;** however, there was no improvement in the symptoms. Physical examination showed a vesicular rash over the left lip and left external auditory canal (scabbed), as shown in Figures 1A and 1B, decreased sweating on the left side of the body, except the face, as shown in Figures 2A and 2B, soft neck, Kernig’s sign (-) and Brudzinski’s sign (-). Mental status examination, cranial nerves, motor exam, reflexes, sensory testing, coordination, and gait were normal.

We conducted a Minor's iodine-starch test, which showed reduced sweating in the left posterior back and left popliteal fossa (Fig. 2). The Minor's iodine-starch test was performed by staining the patient with an iodine solution (1.5 g iodine, 10 g castor oil, and 125 ml 95% ethanol) [6]. Head magnetic resonance imaging (MRI) with contrast showed no abnormalities. Based on the sore throat, dizziness, fever, reduced sweating on the left side of the body, and herpes infection on the left upper lip and left external ear canal, we considered the possibility of VZV meningitis; therefore, a lumbar puncture was performed.

The cerebral spinal fluid (CSF) opening pressure was 105 mmH_2_O. Routine and biochemical testing of CSF identified the following: protein 0.17 g/l (0.20–0.40); leukocytes 26 × 10^6^ /l (0.0–15.0); glucose 58.5 mg/dl (45–81);. CSF cytology was mainly characterized by lymphocyte reactions; we did not find atypical cells, ruling out lymphoma. CSF pathogenic examination showed a negative CSF culture. Next-generation sequencing (NGS) of CSF was used for the detection of pathogens. After 48 h, the results revealed 71 sequence reads uniquely corresponding to the VZV genome with 3.5209% coverage (Fig. 3).

Sympathetic skin response (SSR): The latency wave amplitudes of both hands and feet were within the normal range, and the amplitude of the left foot wave was slightly lower than that of the right side. Stimulating the left hand and left foot wave amplitudes was slightly lower than stimulating the right hand and right foot, showing left side autonomic nerve damage, as shown in Fig. 4.

The patient was diagnosed with viral meningitis by VZV infection and multiple cranial nerve damage (trigeminal and vestibular nerves). We administered 3 g of sodium phosphonate as an antiviral along with B-vitamin nutritional therapy. The role of sodium phosphonate as an antiviral involves blocking the phosphate binding site of viral DNA polymerase in a non-competitive manner to prevent the viral DNA strands extension [7]. After one wk, a repeat lumbar puncture was performed, with the CSF test showing a CSF pressure of 120 mmH_2_O, cerebrospinal fluid white blood cell count of 1 cell/mm^3^, and a CSF protein level of 8 mg/dl. The patient recovered well from the sore throat, with dizziness disappearing and resumption to a normal body temperature. Two months after discharge, the reduced sweating on the left side of the body returned to normal. The one-year follow-up after discharge showed no significant sequelae.

**Discussion and Conclusions**

HZ, also known as zoster, is an infectious skin disease caused by the reactivation of the VZV, which has been dormant in the posterior root ganglia or cranial ganglia of the spinal cord [8]. HZ is a common viral skin infection [9].

In addition to skin damage, HZ is often accompanied by neuropathic pain and is more common in older, immunosuppressed, and immunodeficient populations. It can seriously impact patient quality of life [6], and many patients experience severe physical, occupational, social, and psychosocial disabilities due to persistent pain. Reports in the literature suggest that VZV can also cause HZ in the eyes, which can be complicated by corneal perforation, acute iridocyclitis, vitreous inflammation, necrotizing retinitis, and obstructive retinal vasculitis, potentially leading to retinal detachment, decreased vision, and blindness [10]. HZ is often accompanied by dizziness and changes in taste and hearing. Neurological complications include aseptic meningitis, white matter disease, peripheral motor neuropathy, and Guillain-Barré syndrome [11]. Patients with severe immune deficiency are prone to disseminated HZ and visceral damage, which can manifest as pneumonia, hepatitis, or encephalitis[12].

In this case，we found a VZV infection that caused a decrease in unilateral sweating. This sequela is very rare, and further research is needed on the location and potential mechanisms of its damage. The SSR examination showed a decrease in the left side of wave amplitude, as shown in Fig. 4. This indicated that the patient had autonomic nerve damage on the left side. To further clarify the potential mechanisms of this damage, we examined the literature.

It is known that the VZV lurks in the brain ganglia and spinal dorsal root ganglia, but it can also remain dormant in autonomic ganglia [13], a finding which is rarely documented. In 2001, researchers used polymerase chain reaction (PCR) to detect the DNA of the VZV in human lymph nodes and abdominal ganglia, the first detection of VZV DNA in human autonomic nervous system ganglia [14]. The pathological and physiological mechanisms of autonomic nerve injury remain unclear. Some studies report that reactivation of the virus in the posterior root ganglia and its transmission through affected nerves leads to severe ganglial inflammation and neuritis. This process results in strong sympathetic nerve stimulation and vasoconstriction of small arteries within the nerve, thereby reducing blood flow in the capillary bed within the nerve and causing nerve ischemia [15]. It can therefore be inferred that the virus can reactivate in the autonomic ganglia, spread through the affected nerves, and damage the autonomic nervous system. The patient may have a weakened immune system after catching a cold, and the VZV in the brain ganglia and autonomic ganglia may be activated, damaging the glossopharyngeal, vestibular, trigeminal, autonomic, and retrograde nerve roots entering the cerebrospinal fluid, resulting in the various discomfort symptoms mentioned earlier.

There are few reports of VZV infection complicated by damage to the autonomic nervous system. In 2022, researchers systematically reviewed 45 articles on autonomic dysfunction caused by VZV infection since 1956, including four cases of pupillary dysfunction, two of uterine dysfunction, two of cardiovascular dysfunction, 14 of gastrointestinal dysfunction, and 23 of urinary and reproductive dysfunction [16]. There is only one case reported of reduced sweating caused by VZV infection [17]. However, that study did not confirm autonomic nervous system damage through examination, nor did it mention the potential mechanisms of damage to the autonomic nervous system. With this case report, we demonstrate that VZV infection can lead to unilateral autonomic nervous system damage, and we have attempted to clarify the potential mechanisms of its damage.

Because the symptoms of limb pain may mask the symptoms of reduced lateral sweating, the incidence of secondary reduced lateral sweating in HZ may be underestimated. We aimed to raise general awareness of this symptom. However, VZV infection causing unilateral autonomic nerve damage has rarely been described as a neurological complication of HZ. The significance of this case lies in demonstrating the need for dermatologists to understand and identify the syndrome so that antiviral treatment can be initiated early to avoid other possible serious complications. It should also be of value in multidisciplinary patient management with neurologists and/or otolaryngologists.

In summary, the secondary reduction of unilateral sweating is a rare neurological complication of HZ, and the mechanism of its occurrence has not yet been elucidated. At present, there is a lack of evidence-based medicine for treatment; however, in this report, the patient's antiviral treatment was effective, and the prognosis was relatively good. For patients with acutely reduced sweating, physicians should consider the possibility of secondary autonomic nervous system damage caused by HZ. Future research should investigate the underlying mechanism of the symptoms noted here.

**Abbreviations**

CSF - cerebral spinal fluid

HZ - herpes zoster

MRI - magnetic resonance imaging

NGS - next-generation sequencing

PCR - polymerase chain reaction

RHS - Ramsay Hunt syndrome

SSR - sympathetic skin response

VZV - varicella zoster virus

**DECLARATIONS:**

**Ethics approval and consent to participate**

Written informed consent was obtained from the patient for publication of this case report and any accompanying images. A copy of the written consent is available for review by the Editor of this journal. Since no human experimentation was performed, no approval by an ethics board was required.

**Consent for publication**

Written informed consent for publication was obtained from the patient. Consent to publish refers to the use of identifiable materials, including photos.

**Availability of data and materials**

We declare that the materials described in the manuscript, including all relevant raw data, will be freely available to any scientist wishing to use them for non-commercial purposes without breaching participant confidentiality.

**Competing interests**

The authors declare that they have no competing interests.

**Funding**

This research received no specific grant from any funding agency in the public, commercial, or not-for-profit sectors.

**Authors’ contributions**

WL and BL treated the patient and wrote the draft of the manuscript. JZ, YZ, and LY collected, analyzed, and interpreted the data. KH and CL also treated the patient and critically revised the manuscript. All authors provided their final approval for the version to be published. All the authors have read and approved the final version of the manuscript.

**Acknowledgments**

We thank the physicians who provided the clinical support. We also thank the family of this patient. We thank Editage [http://www.editage.cn] for the English language editing.

**References**

References

1. Bienkowski C, Kowalczyk M, Talarek E, Pokorska-Spiewak M, Kierdaszuk B, Marczynska M. Meningitis and Ramsay-Hunt syndrome in a 17-year old girl. Neuro Endocrinol Lett. 2019;40(3):149-51. PMID [31816219](https://www.ncbi.nlm.nih.gov/pubmed/31816219).

2. Steiner I, Kennedy PG, Pachner AR. The neurotropic herpes viruses: herpes simplex and varicella-zoster. Lancet Neurol. 2007;6(11):1015-28. doi: [10.1016/S1474-4422(07)70267-3](https://doi.org/10.1016/S1474-4422(07)70267-3), PMID [17945155](https://www.ncbi.nlm.nih.gov/pubmed/17945155).

3. Lee JH, Kang J, Seo YD, Kim HC, Shin KJ. Delayed facial palsy in a patient with varicella zoster virus encephalitis. J Clin Neurol. 2021;17(4):604-6. doi: [10.3988/jcn.2021.17.4.604](https://doi.org/10.3988/jcn.2021.17.4.604), PMID [34595877](https://www.ncbi.nlm.nih.gov/pubmed/34595877).

4. Chamizo FJ, Gilarranz R, Hernández M, Ramos D, Pena MJ. Central nervous system infections caused by varicella-zoster virus. J Neurovirol. 2016;22(4):529-32. doi: [10.1007/s13365-016-0422-y](https://doi.org/10.1007/s13365-016-0422-y), PMID [26769041](https://www.ncbi.nlm.nih.gov/pubmed/26769041).

5. Aviel A, Marshak G. Ramsay Hunt syndrome: a cranial polyneuropathy. Am J Otolaryngol. 1982;3(1):61-6. doi: [10.1016/s0196-0709(82)80035-5](https://doi.org/10.1016/s0196-0709(82)80035-5), PMID [6981355](https://www.ncbi.nlm.nih.gov/pubmed/6981355).

6. Minor V. Ein neues Verfahren zu der klinischen Untersuchung der Schweißabsonderung. Deutsche Zeitschrift f Nervenheilkunde. 1928;101(1):302-8. doi: [10.1007/BF01652699](https://doi.org/10.1007/BF01652699).

7. Nikkels AF, Schoevaerdts D, Kauffmann F, Strubbe F, Bensemmane S. Herpes zoster in Belgium: a new solution to an old problem. Acta Clin Belg. 2024:1-12. doi: [10.1080/17843286.2024.2350258](https://doi.org/10.1080/17843286.2024.2350258), PMID [38781037](https://www.ncbi.nlm.nih.gov/pubmed/38781037).

8. Sauerbrei A. Diagnosis, antiviral therapy, and prophylaxis of varicella-zoster virus infections. Eur J Clin Microbiol Infect Dis. 2016;35(5):723-34. doi: [10.1007/s10096-016-2605-0](https://doi.org/10.1007/s10096-016-2605-0), PMID [26873382](https://www.ncbi.nlm.nih.gov/pubmed/26873382).

9. Ultsch B, Köster I, Reinhold T, Siedler A, Krause G, Icks A et al. Epidemiology and cost of herpes zoster and postherpetic neuralgia in Germany. Eur J Health Econ. 2013;14(6):1015-26. doi: [10.1007/s10198-012-0452-1](https://doi.org/10.1007/s10198-012-0452-1), PMID [23271349](https://www.ncbi.nlm.nih.gov/pubmed/23271349).

10. Ting DS, Ghosh N, Ghosh S. Herpes zoster ophthalmicus. BMJ. 2019;364:k5234. doi: [10.1136/bmj.k5234](https://doi.org/10.1136/bmj.k5234), PMID [30655269](https://www.ncbi.nlm.nih.gov/pubmed/30655269).

11. Gross G. [Zoster. The manifestation forms in the skin, complications and therapy]. Dtsch Med Wochenschr. 1997;122(5):132-9. doi: [10.1055/s-2008-1047587](https://doi.org/10.1055/s-2008-1047587), PMID [9072484](https://www.ncbi.nlm.nih.gov/pubmed/9072484).

12. Stratman E. Visceral zoster as the presenting feature of disseminated herpes zoster. J Am Acad Dermatol. 2002;46(5):771-4. doi: [10.1067/mjd.2002.119091](https://doi.org/10.1067/mjd.2002.119091), PMID [12004322](https://www.ncbi.nlm.nih.gov/pubmed/12004322).

13. Mueller NH, Gilden DH, Cohrs RJ, Mahalingam R, Nagel MA. Varicella zoster virus infection: clinical features, molecular pathogenesis of disease, and latency. Neurol Clin. 2008;26(3):675-97, viii. doi: [10.1016/j.ncl.2008.03.011](https://doi.org/10.1016/j.ncl.2008.03.011), PMID [18657721](https://www.ncbi.nlm.nih.gov/pubmed/18657721).

14. Gilden DH, Gesser R, Smith J, Wellish M, Laguardia JJ, Cohrs RJ et al. Presence of VZV and HSV-1 DNA in human nodose and celiac ganglia. Virus Genes. 2001;23(2):145-7. doi: [10.1023/a:1011883919058](https://doi.org/10.1023/a:1011883919058), PMID [11724266](https://www.ncbi.nlm.nih.gov/pubmed/11724266).

15. Makharita MY. Prevention of post-herpetic neuralgia from dream to reality: A ten-step model. Pain Phys. 2017;20(2):E209-20. PMID [28158158](https://www.ncbi.nlm.nih.gov/pubmed/28158158).

16. Sakakibara R, Sawai S, Ogata T. Varicella-zoster virus infection and autonomic dysfunction. Auton Neurosci. 2022;242:103018. doi: [10.1016/j.autneu.2022.103018](https://doi.org/10.1016/j.autneu.2022.103018), PMID [35863181](https://www.ncbi.nlm.nih.gov/pubmed/35863181).

17. Kurihara K, Tsushima T, Tokura Y. Idiopathic segmental anhidrosis associated with varicella. J Dermatol. 2017;44(2):211-2. doi: [10.1111/1346-8138.13455](https://doi.org/10.1111/1346-8138.13455), PMID [27206820](https://www.ncbi.nlm.nih.gov/pubmed/27206820).

**Figure legends**

**Fig. 1**: Multiple blisters (scabbed) are visible on the skin of the patient's left upper lip (A) and left external ear canal (B).

**Fig. 2**: We conducted an improved Minor's iodine-starch test, which showed sweating in the right back and reduced sweating in the left back (A), and sweating in the right popliteal fossa and reduced sweating in the left popliteal fossa (B).

**Fig. 3**: Head MRI with contrast showed no abnormalities.

**Fig. 4**: mNGS of Human alphaherpesvirus-3 in the patient's cerebrospinal fluid (A), and

(B) the result of mNGS showed 71 reads corresponding to the human alphaherpesvirus-3, with a coverage of 3.5209%. In the microbiota composition, 0.05% of the reads corresponded to human alphaherpesvirus-3, and the remainder are commonly regarded as contaminating bacterial DNA from the environment.

**Fig. 5**: SSR shows the amplitude of the left foot is slightly lower than that of the right foot, while the amplitude of the left hand and left foot is slightly lower than that of the right hand and foot.Conclusion: SSR indicates damage to the left sympathetic nerve.
